# The ubiquitin-like molecule interferon-stimulated gene 15 (ISG15) is a potential prognostic marker in human breast cancer

**DOI:** 10.1186/bcr2117

**Published:** 2008-07-15

**Authors:** Nuran Bektas, Erik Noetzel, Jürgen Veeck, Michael F Press, Glen Kristiansen, Amjad Naami, Arndt Hartmann, Arno Dimmler, Matthias W Beckmann, Ruth Knüchel, Peter A Fasching, Edgar Dahl

**Affiliations:** 1 Department of Pathology, University Hospital of the RWTH Aachen, Pauwelsstrasse 30, 52074 Aachen, Germany; 2 Department of Pathology, Keck School of Medicine, University of Southern California/Norris Comprehensive Cancer Center, 1441 Eastlake Avenue, Los Angeles, CA 90033, USA; 3 Department of Pathology, University Hospital Zürich, Schmelzbergstrasse 12, 8091 Zürich, Switzerland; 4 Department of Pathology, University Hospital Erlangen, Krankenhausstrasse 12, 91054 Erlangen, Germany; 5 Department of Gynaecology, University Hospital Erlangen, Universitätsstrasse 21-23, 91054 Erlangen, Germany

## Abstract

**Introduction:**

*ISG15 *is an ubiquitin-like molecule that is strongly upregulated by type I interferons as a primary response to diverse microbial and cellular stress stimuli. However, alterations in the *ISG15 *signalling pathway have also been found in several human tumour entities. To the best of our knowledge, in the current study we present for the first time a systematic characterisation of *ISG15 *expression in human breast cancer and normal breast tissue both at the mRNA and protein level.

**Method:**

Using semiquantitative real-time PCR, cDNA dot-blot hybridisation and immunohistochemistry, we systematically analysed *ISG15 *expression in invasive breast carcinomas (*n *= 910) and normal breast tissues (*n* = 135). *ISG15 *protein expression was analysed in two independent cohorts on tissue microarrays; in an initial evaluation set of 179 breast carcinomas and 51 normal breast tissues; and in a second large validation set of 646 breast carcinomas and 10 normal breast tissues. In addition, a collection of benign and malignant mammary cell lines (*n* = 9) were investigated for *ISG15 *expression.

**Results:**

*ISG15 *was overexpressed in breast carcinoma cells compared with normal breast tissue, both at the RNA and protein level. Recurrence-free (*p *= 0.030), event-free (*p *= 0.001) and overall (*p *= 0.001) survival analyses showed a significant correlation between *ISG15 *overexpression and unfavourable prognosis.

**Conclusion:**

Therefore, *ISG15 *may represent a novel breast tumour marker with prognostic significance and may be helpful in selecting patients for and predicting response to the treatment of human breast cancer.

## Introduction

The interferon-stimulated gene 15 kDa (*ISG15*) is an interferon-inducible ubiquitin-like protein. In addition to being stimulated by type I interferon, expression of *ISG15 *is greatly induced by viral or bacterial infection through the Janus kinase/signal transducer and activator of transcription (*Jak*/STAT) signalling pathway [1-3]. After induction, *ISG15 *is secreted by monocytes, B- and T-lymphocytes and fibroblasts. The *ISG15 *protein is made up of tandem ubiquitin homology domains [4]. Within cells, *ISG15 *is covalently conjugated with cellular proteins in an enzymatic pathway (so called ISGylation) comprised of the activating E1, conjugating E2 and ligating E3 enzymes, which are similar to those used by ubiquitin (ubiquitylation). The conjugation of *ISG15 *with protein substrates provides a tag that either marks the labelled protein for degradation [5] or modulates its function [6]. Importantly, protein ISGylation is a reversible process, and removal of *ISG15 *molecules is mediated by de-ISGylating enzymes [7].

Multiple target proteins of *ISG15 *have recently been identified, some of which have been implicated in the regulation of immune responses. However, the biological consequences of modification by *ISG15 *need to be determined further [8]. Interestingly, protein ubiquitylation has been shown to be of fundamental importance in the regulation of both the innate and adaptive immune systems, with roles in the control of immune tolerance, the differentiation of T-cells and the intracellular signal transduction induced by antigen, cytokines or toll-like receptor ligands [9].

So far, little is known about the function of *ISG15 *in carcinogenesis. *ISG15 *is a post-translational protein modifier and also a secreted cytokine. Each of these roles has been implicated in carcinogenesis, although *ISG15 *was recently thought to function as an oncogene as well as a tumour-suppressor gene [10]. Enhanced ISGylation was observed in response to cancer chemotherapeutics, suggesting that *ISG15 *has a tumour-suppressing function. Moreover, *ISG15 *is a target of the tumour-suppressor gene *TP53 *[11]. In contrast, deregulated overexpression of *ISG15 *and enhanced ISGylation were positively correlated with cancerogenesis underlining the oncogenic potential of *ISG15 *[12-14]. As a secreted protein, *ISG15 *has been shown to primarily modulate immune cell activation [15].

Some of the *ISG15 *target proteins also have functions in the cell nucleus including chromatin remodelling and polymerase II transcription [16]. Interestingly, in bladder cancer immunohistochemistry revealed that *ISG15 *was located predominantly in the nuclei of cancer cells which were associated with an advanced tumour stage [12]. Surprisingly, *ISG15 *overexpression in human bladder cancer has not been found to be associated with a general inflammatory response even though *ISG15 *was expected to be stimulated by interferon primarily in an inflammatory process. Therefore, *ISG15 *expression in bladder cancer suggests that it is specifically associated with tumour cells [12].

In breast cancer, *ISG15 *expression has so far only been examined in benign and malignant human breast cell lines [13]. To the best of our knowledge, this is the first study to systematically analyse the expression of *ISG15 *in human breast carcinomas and normal breast tissues both at the mRNA and the protein level. We analysed the results particularly in correlation to clinicopathological data, such as hormone receptor status, *HER2 *status and patient survival data, in order to validate *ISG15 *as a new prognostic marker and potential drug target in the treatment of human breast cancer.

## Materials and methods

*ISG15 *protein expression was assessed in two independent cohorts of breast cancer patients using tissue microarrays (TMAs). The first tumour cohort has been previously described [17] and consisted of 179 breast cancer tissue specimens and 51 normal breast tissue specimens. The TMA used one tissue core from non-selected, formalin-fixed, paraffin-embedded primary breast cancer specimens from patients diagnosed between 1994 and 2002 at the Institute of Pathology, University of Regensburg, Germany. The patients' ages ranged from 25 to 82 years with a median age of 56 years. An experienced surgical pathologist (AH) evaluated slides of all specimens, stained with haematoxylin and eosin (H&E), before construction of the TMA in order to identify representative tumour areas. Histologically, all tumours were graded according to criteria by Elston and Ellis [18]. Clinical follow-up data, provided by the German Central Tumour Registry were available for all 179 breast cancer patients with a median follow-up period of 78 months (range 0 to 148 months). The Institutional Review Board of the participating centres approved the study.

The second TMA for validation consisted of 967 breast specimens, of which it was possible to analyse 646 breast carcinomas and 10 normal breast tissues samples. The second TMA was constructed from a cohort of breast cancer patients who were participants of a case-controlled trial for the assessment of breast cancer susceptibility markers and prognostic factors, the Bavarian Breast Cancer Cases and Controls (BBCC), which has been described previously, including methods for the data collection [19,20]. The database for this analysis closed on 31 December, 2007 and had a median follow up of 4.7 years. Paraffin embedded tissue was available for 967 BBCC patients. Briefly a 0.6 mm punch was retrieved from the breast cancer tumour after a pathologist (AD) evaluated H&E-stained slides of all specimens. Data about the hormone receptor status were obtained from original pathological reports of these specimens; the other data were collected from the patients' medical records.

The collection of paraffin-embedded and formalin-fixed normal (*n* = 14) and cancerous *(n *= 25) breast tissue samples for the mRNA expression analysis has been described previously [21].

### Cell lines

The human mammary epithelial cell lines HMEC and MCF12A, and the breast cancer cell lines MCF7, T47D, ZR75-1, MDA-MB231, MDA-MB468, SKBR3 and BT20 were obtained from the ATCC (Rockville, MD, USA) and cultured as previously described [22].

### RNA extraction and reverse transcription

Total RNA was isolated using the TRIzol reagent (Invitrogen, Carlsbad, CA, USA) according to the manufacturer's recommendations. For paraffin-embedded tissues, five consecutive sections (each 5 μm thick) were prepared, deparaffinised and conventionally re-hydrated in a decreasing alcohol-series before RNA extraction. Of the obtained RNA, 1 μg was reverse transcribed using the Reverse Transcription System (Promega, Madison, WI, USA). In order to improve the transcription rate oligo-dT was mixed with pdN_6 _primers at a ratio of 1:2.

### Semiquantitative real-time PCR

Semiquantitative PCR was performed using the LightCycler system with the LightCycler DNA Master SYBR Green I Kit (Roche Diagnostics, Mannheim, Germany) as previously described [22]. To ensure the accuracy of the experiment, all reactions were performed in triplicate. Primer sequences used were:*ISG15 *sense 5'- GAG AGG CAG CGA ACT CAT CT -3' and antisense 5'- CTT CAG CTC TGA CAC CGA CA -3'; *GAPDH *sense 5'- GAA GGT GAA GGT CGG AGT CA -3' and antisense 5'- AAT GAA GGG CTC ATT GAT GG -3'. The annealing temperature for both genes was 60°C. Reaction specificity was controlled by post-amplification melting curve analyses and gel electrophoresis of the obtained products.

### Breast cancer cDNA dot-blot hybridisation

The breast cancer profiling array (BD Clontech, Heidelberg, Germany) contains 50 pairs of cDNAs generated from matched tumourous and normal breast tissue samples from individual patients and three breast cancer lymph node metastasis specimens, spotted on a nylon membrane [23]. Hybridisations using 25 ng of a gene-specific ^32^P-labelled cDNA probe digested from Unigene cDNA clones were conducted according to the manufacturer's recommendations. The tumour/normal intensity ratio was calculated using a STORM-860 phosphoimager (Molecular Dynamics, Sunnyvale, CA, USA) and normalised against the background.

Immunohistochemical studies for the expression of *HER2 *utilised an avidin-biotin peroxidase method with a 3,3'-diaminobenzidine chromatogen. After antigen retrieval (microwave oven for 30 minutes at 200W) immunohistochemistry studies were performed in a NEXES immunostainer (Ventana, Tucson, AZ, USA) according to the manufacturer's instructions. The primary antibodies used were anti-*HER2 *(DAKO, Hamburg, Germany; 1:400), anti-oestrogen receptor (ER) and anti-progesterone receptor (PR) (Novocastra, Newcastle Upon Tyne, UK; 1:20). For target proteins, the ChemMate detection kit (DAKO, Hamburg, Germany) was used. A surgical pathologist (AH) performed a blinded evaluation of the TMA slides with no knowledge of clinical data. It was not possible to interpret some results for reasons that included a lack of tumour tissue and the presence of necrosis or crush artefacts. *HER2 *expression was scored according to the DAKO HercepTest. For the evaluation of the presence of ER and PR, a semiquantitative immunoreactivity score (IRS), as described by Remmele and Stegner [24], was used.

### *ISG15 *Immunohistochemistry

The TMAs of both the evaluation set and the validation set were subjected to immunostaining using the Advance Kit (K4068, DAKO, Hamburg, Germany) following the manufacturer's instructions. Paraffin-embedded breast carcinomas were used as positive controls. After deparaffinisation and rehydration the tissue samples were heated in a microwave oven for 30 minutes at 200W in 10 mM sodium citrate buffer (pH 7.2). Endogenous peroxidase was blocked by peroxidase-blocking solution (S2023, DAKO, Hamburg, Germany) for 10 minutes. The polyclonal primary antibody *ISG15 *(AP 1150a, rabbit, Abgent, San Diego, CA, USA) was applied (1:30 dilution) for 30 minutes at room temperature. The primary antibody was omitted in the negative controls. For signal detection 3,3'-diaminobenzidine was used. Slides were counterstained with H&E and after dehydration mounted in Vitro-Clud (Langenbrinck, Emmendingen, Germany). Two experienced pathologists (NB, AN) scored the immunohistochemical staining intensity according to the scoring system suggested by Remmele and Stegner [24].

### Statistical methods

For statistical evaluation SPSS version 14.0 (SPSS Inc, Chicago, IL) was used. Differences were considered statistically significant when p < 0.05. A non-parametrically two-tailed Mann-Whitney *U*-test was employed to analyse differences in expression levels. For analysis of the cancer profiling array, a Kolmogorov-Smirnov test was applied to test for a normal value distribution, followed by a two-sided paired *t*-test to analyse differences in normal and tumour expression. A statistical association between clinicopathological and molecular parameters was tested for using a two-sided Fisher's exact test. Recurrence-free survival (RFS) and overall survival (OS) were calculated according to the Kaplan-Meier equation.

For the BBCC-TMA, RFS was defined as any local recurrence or distant recurrence, whatever occurred first. Event-free survival (EFS) was defined as any local recurrence, distant recurrence or death, whatever occurred first. Kaplan-Meier estimates were used to display the survival curves and log-rank test was used to compare patients with high vs. low *ISG15 *expression. For multivariate analyses, a Cox proportional hazard model was constructed for OS, EFS and RFS.

## Results

### Upregulation of *ISG15 *in human breast cancer cell lines

**Figure 1:**
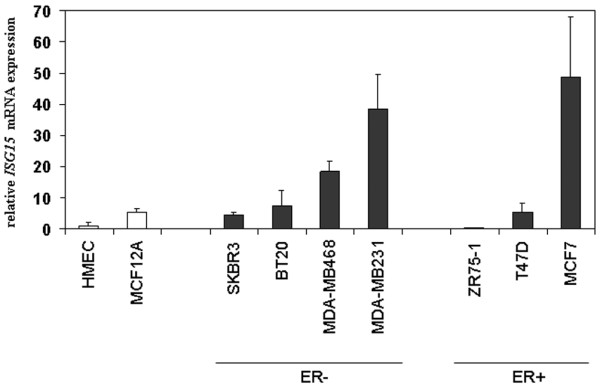
Abundance of the expression of *ISG15 *mRNA in breast cancer cell lines. Semiquantitative real-time PCR (LightCycler) of *ISG15 *expression was performed on reverse transcribed RNA from non-malignant (white bars) and malignant cell lines (black bars). *ISG15 *mRNA expression was low among the non-malignant mammary epithelial cell lines HMEC and MCF12A, whereas *ISG15 *mRNA transcript levels varied more and in summary were elevated in the investigated cancerous cell lines.

We started the study by investigating the level of *ISG15 *mRNA expression in non-malignant and malignant human mammary cell lines. Real-time PCR analysis revealed a low *ISG15 *mRNA expression among the non-malignant mammary epithelial cell lines HMEC and MCF12A, whereas *ISG15 *mRNA transcript levels varied more and in summary were clearly elevated in the cancerous cell lines BT20, MDA-MB468, MDA-MB231, T47D and MCF7, of which MCF7 displayed an exceptionally high level of *ISG15 *mRNA (Figure 1). The median elevation of *ISG15 *expression in malignant cell lines vs. non-malignant cell lines was 2.5-fold and these differences in expression between the respective groups were statistically significant (*p *= 0.043, Mann-Whitney *U*-test). Differences in expression levels between ER-positive and ER-negative breast cancer cell lines were statistically not significant.

### Upregulation of *ISG15 *in primary breast cancers analysed by real-time PCR

**Figure 2:**
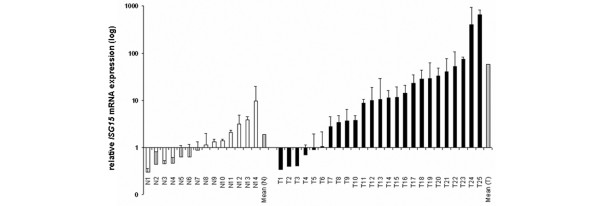
Upregulation of *ISG15 *mRNA in primary breast cancer. A collection of paraffin-embedded breast carcinomas (T; *n* = 25) and normal breast tissues (N; *n* = 14) was analysed for *ISG15 *expression by semiquantative real-time PCR. *ISG15 *was strongly upregulated at the transcript level in the tumourous tissues compared with the normal breast tissues.

Next we analysed *ISG15 *mRNA expression in primary breast cancers (*n* = 25) and normal mammary samples (*n* = 14) derived from formalin-fixed, paraffin-embedded tissues by real-time PCR. In line with our breast cell line data, *ISG15 *mRNA was abundantly expressed in primary breast cancer specimens when compared with normal mammary epithelial tissue (Figure 2). The median fold change of *ISG15 *upregulation in the cancerous tissue vs. normal tissue was 10.3 and differences in expression between the two groups were statistically significant (*p *= 0.002, Mann-Whitney *U*-test).

### Correlation between *ISG15 *mRNA expression and protein expression

**Figure 3:**
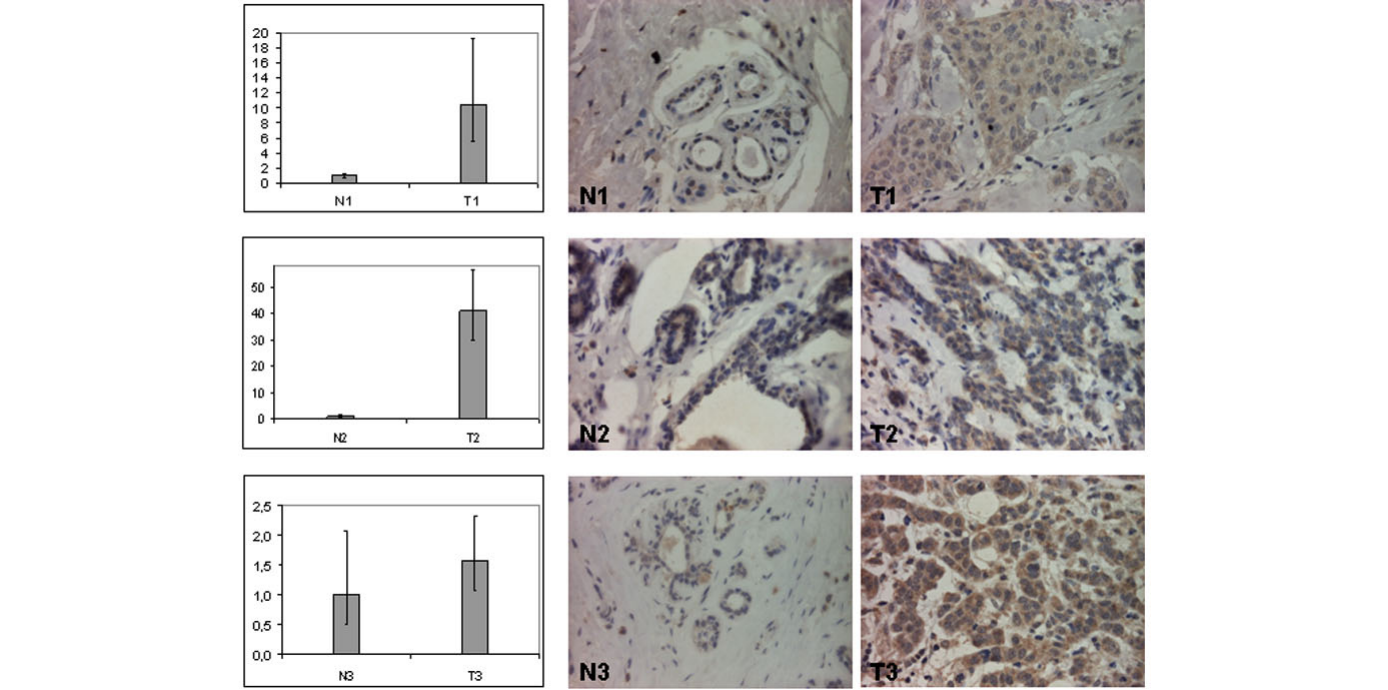
Correlation between *ISG15 *mRNA and protein expression. An additional collection of paraffin-embedded breast carcinomas (T; *n* = 10) and corresponding normal breast tissues (N; *n* = 10) was analysed for *ISG15 *expression by semiquantitative real-time PCR and immunohistochemistry in the same samples. *ISG15 *mRNA upregulation correlated with *ISG15 *protein expression in the same tumour sample (T) compared with the corresponding normal breast tissue (N). Magnification: 400×.

We additionally analysed 10 paraffin-embedded breast carcinomas and corresponding normal breast tissues by both semiquantitative real-time PCR and immunohistochemistry in order to correlate mRNA and protein levels in the same tumour samples as well as in the corresponding normal breast tissues. *ISG15 *mRNA was significantly upregulated in breast cancer specimens when compared with the corresponding normal breast tissues (*p *= 0.003, Mann-Whitney *U*-test). The median change in the tumour was three-fold compared with normal breast tissue on *ISG15 *mRNA level. At the protein level as determined by immunohistochemistry *ISG15 *was overexpressed in the tumour and correlated with upregulation of *ISG15 *mRNA. The median IRS in the tumour was five compared with an IRS of two in normal breast tissue and differences between the groups were statistically significant (p < 0.01, Mann-Whitney *U*-test) (Figure 3).

### Upregulation of *ISG15 *in primary breast cancer analysed by cDNA dot-blot hybridisation

**Figure 4:**
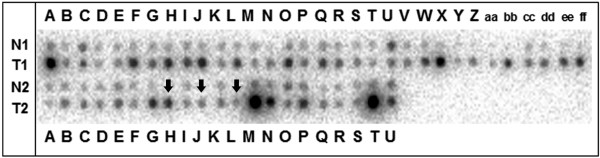
Expression profiles were determined using cDNA dot-blot hybridisation analysis (BD Clontech) containing cDNA pairs derived from 50 matched normal breast tissues (N), tumourous breast tissues (T) and three lymph node metastastic tissues (marked with arrows). Upregulation of *ISG15 *was observed in primary breast tumours and in one of three metastatic lymph nodes, as compared with matched normal breast tissue.

*ISG15 *upregulation was validated by dot-blot analysis on a nylon array containing spotted cDNAs derived from 50 matched pairs of normal and cancerous breast tissue (Figure 4). The cancer profiling array showed upregulation (fold change ≥ 2) of *ISG15 *in 33 of 50 primary breast tumours (66%), as well as in one of three metastatic lymph nodes, compared with matched normal breast tissue (two-sided paired *t*-test: *p *< 0.001). Mean densitometric intensity in normal tissues was 1533 arbitrary units (standard deviation [SD] ± 995) and 3571 arbitrary units (SD ± 2292) in tumour tissues.

### Overexpression of *ISG15 *protein in primary breast carcinomas

**Figure 5:**
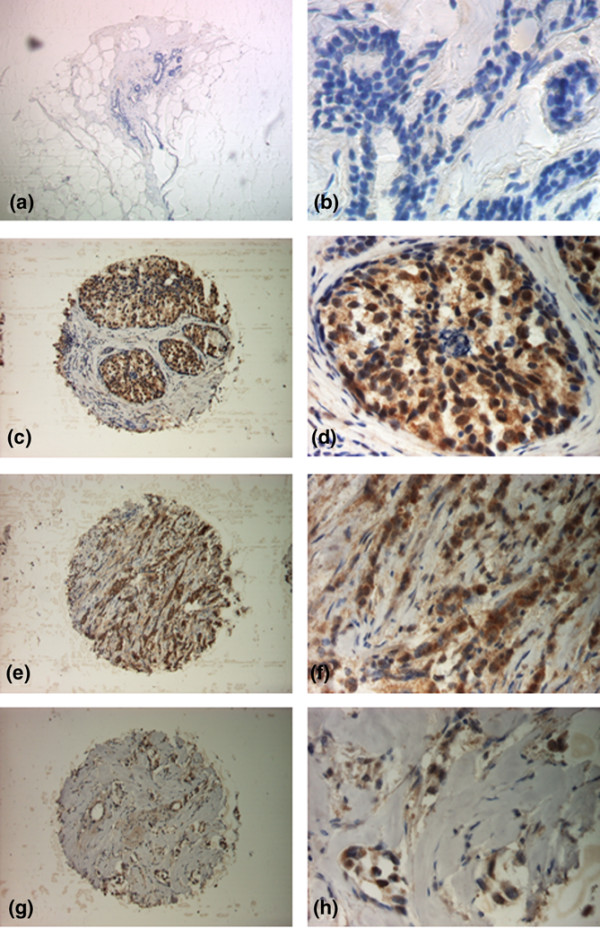
Immunohistochemical expression of *ISG15 *in normal breast tissue, and in non-invasive and invasive breast tumours using a tissue microarray. (a, b) Normal breast tissue lacks *ISG15 *expression (IRS = 0). (c, d) In ductal carcinoma *in situ ISG15 *expression is more intense (IRS = 8) than in normal breast tissue. (e, f) In invasive breast carcinomas (here: ductal type) *ISG15 *expression was clearly more abundant (example shows staining with an IRS = 12) compared with normal breast tissue and ductal carcinoma *in situ*. (g, h) In tubular breast carcinomas (IRS = 4), a less frequent variant of invasive breast carcinomas with a more favourable prognosis than invasive ductal breast carcinomas, *ISG15 *expression was less abundant than in most invasive ductal breast carcinomas and stronger than in most normal breast tissues. Magnifications: a, c, e, g: 100×; b, d, f, h: 400×.

Two independent cohorts of breast cancer specimens arranged on two different TMAs were analysed for *ISG15 *expression by immunohistochemistry. These represented an initial evaluation set, consisting of 179 breast carcinomas and 51 normal breast tissues, and an independent validation set, consisting of 646 breast carcinomas and 10 normal breast tissues. In normal breast tissue *ISG15 *expression was often absent or weak (Figure 5a, b). In ductal carcinoma *in situ *(Figure 5c, d) *ISG15 *expression was more intense than in normal breast tissue. In invasive breast carcinomas (Figure 5e, f, ductal type) *ISG15 *expression was generally more abundant than in either ductal carcinoma *in situ *or normal breast tissue. In tubular breast carcinomas (Figure 5g, h), a less frequent variant of invasive breast carcinomas with a more favourable prognosis than invasive ductal breast carcinomas, *ISG15 *expression was less abundant than in most invasive ductal breast carcinomas.

### Statistical analysis of the initial evaluation TMA cohort

Statistical data of the initial TMA cohort are presented in Additional files 1, 2, 3, 4. In the initial TMA cohort, *ISG15 *protein expression in breast carcinomas (IRS > 4) was associated with the ER status (*p *= 0.028), but not with tumour size, lymph node status, histological grading, focality or histological type of tumour (Additional file 3). To investigate a possible impact of *ISG15 *overexpression on clinical outcome we calculated univariate survival probability curves with respect to immunohistochemical results. We found that *ISG15 *expression in breast cancer (IRS > 4) showed an unfavourable prognosis with regard to RFS as shown by Kaplan-Meier analysis (*p *= 0.012) (Additional file 1, Additional file 4). Patients who showed abundant *ISG15 *expression in the tumour had an estimated mean RFS of 112 months (95% confidence interval [CI]: 99 to 125) compared with 78 months (95% CI: 68 to 87) in patients with negative/weak *ISG15 *expression. Although OS was not significantly associated with *ISG15 *overexpression, there was a trend towards unfavourable prognosis in those patients with strong *ISG15 *expression (Additional file 2, Additional file 4). Possibly due to a weak association with the ER status, *ISG15 *expression could not be identified as an independent prognostic factor in a multivariate analysis in this breast cancer cohort.

### Validation in the Bavarian breast cancer cases and controls tissue microarray cohort

**Figure 6:**
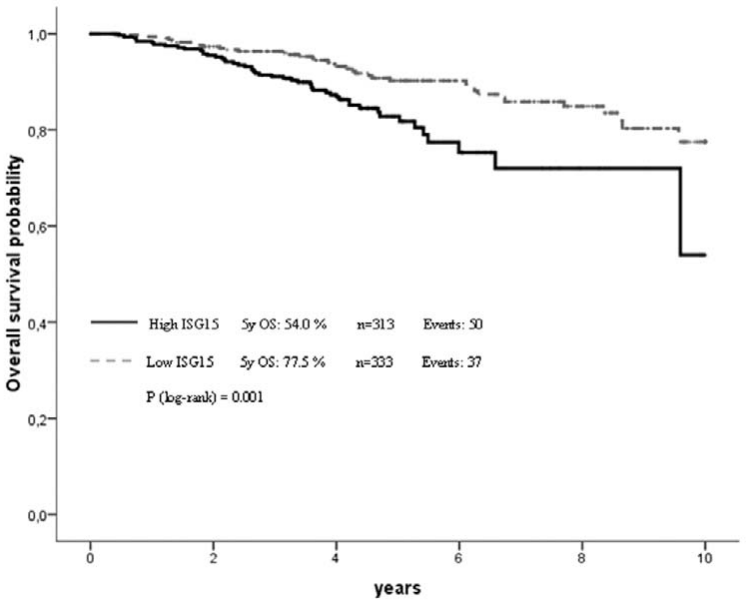
Correlation of *ISG15 *expression and patient prognosis according to univariate Kaplan-Meier analysis. Breast cancer patients overexpressing *ISG15 *show unfavourable prognosis in overall survival analysis (*p *= 0.001).

**Figure 7:**
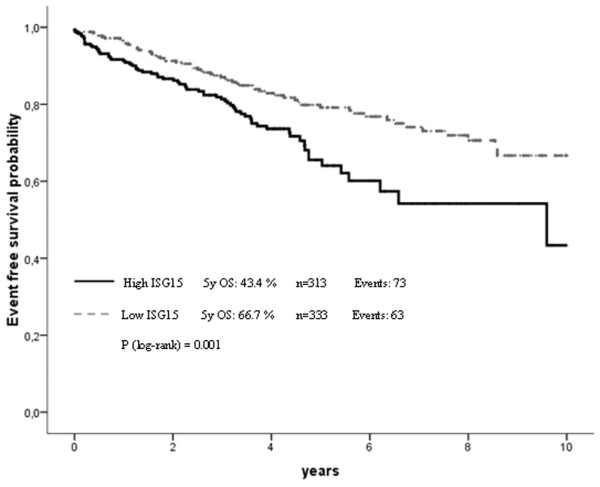
Correlation of *ISG15 *expression and patient prognosis according to univariate Kaplan-Meier analysis. Breast cancer patients overexpressing *ISG15 *exhibit unfavourable prognosis in event-free survival (*p *= 0.001).

To confirm our results from the initial evaluation set of tumours, we analysed a second larger cohort (from BBCC) of breast cancer specimens on a TMA. In this validation cohort the IRS score was available for 646 invasive breast cancer cases. The mean IRS score was 3.5 with 83.9% (*n* = 542) of tumours having a low score of zero to four and 16.1% (*n* = 104) showing abundant expression of *ISG15 *(IRS > 4). As in the initial set we found a close association between *ISG15 *expression and an unfavourable prognosis (that is hazard ratio (HR) = 1.94; 95% CI: 1.17 to 3.23; *p *= 0.01 in OS analysis). However, this cohort of tumours was divided into two groups of similar size for efficient correlation analysis. Therefore, the validation set of the additional 646 breast tumours was divided into low *ISG15 *expressers (IRS = 0 to 3; *n *= 333) and high *ISG15 *expressers (IRS = 4 to 12; *n* = 313). Under this slightly different cut-off level (IRS > 3), the prognostic value of *ISG15 *expression was improved, as *ISG15 *immunohistochemical expression was also an independent predictor for OS and EFS in multivariate analysis (Table 1 and Figures 6 and 7). Furthermore, under this cut-off level, clear associations could be seen between *ISG15 *expression and both tumour size and grading (Table 2).

**Table 1:** Cox proportional hazard ratio analysis of the validation set (variables were categorised as indicated in Table 2)

		*n*	Overall Survival Adjusted hazards ratio (95% CI)	*p*-value	Event-free survival Adjusted hazards ratio (95% CI)	*p*-value	Recurrence-free survival Adjusted hazards ratio (95% CI)	*p*-value
**Age**	< 50	153	1		1		1	
	50–59	154	1.19 (0.54 to 2.63)	0.659	1.15 (0.67 to 1.96)	0.622	1.20 (0.69 to 2.09)	0.516
	60–69	158	1.45 (0.71 to 2.97)	0.309	0.99 (0.59 to 1.67)	0.978	0.81 (0.46 to 1.43)	0.475
	≥ 70	106	3.43 (1.76 to 6.69)	**0.0003**	1.94 (1.17 to 3.22)	**0.010**	1.17 (0.64 to 2.14)	0.612
								
**pT**^a^	T1	297	1		1		1	
	T2-4	274	3.56 (1.89 to 6.67)	**0.00008**	2.88 (1.85 to 4.50)	**0.000003**	3.258 (1.95 to 5.43)	**0.000006**
								
**pN**^a^	0	335	1		1		1	
	1	236	2.81 (1.64 to 4.82)	**0.0002**	2.149 (1.43 to 3.23)	**0.0002**	2.52 (1.57 to 4.04)	**0.0001**
								
**Grading**	1 or 2	385	1		1		1	
	3	186	1.39 (0.82 to 2.36)	0.218	1.05 (0.69 to 1.59)	0.832	1.17 (0.73 to 1.88)	0.513
								
**Hormone receptor status**	Negative	123	1		1		1	
	Positive	448	0.55 (0.32 to 0.95)	**0.032**	0.726 (0.47 to 1.13)	0.160	0.77 (0.46 to 1.26)	0.287
								
***ISG15***	Low (IRS 0 to 3)	277	1		1		1	
	High (IRS 4 to 12)	294	1.78 (1.07 to 2.95)	**0.025**	1.516 (1.03 to 2.22)	**0.033**	1.39 (0.90 to 2.13)	0.135

^a^According to Sobin LH, Wittekind CH: UICC: TNM Classification of Malignant Tumours. 6^th ^edition, New York: Wiley; 2002. [36]. CI, Confidence interval

**Table 2:** Association of *ISG15 *immunoreactivity with clinical and pathological characteristics assessed in clinical routine examination of the validation set

**Characteristic**		**Low *ISG15 *(Score 0 to 3)**	**High *ISG15 *(Score 4 to 12)**	**Total *n* (%)**	***p*-value**
**Total**		333 (51.5)	313 (48.5)	646 (100)	n/a

**Age (*t*-test)**	Mean (± SD)	57.3(± 12.2)	59.2 (± 12.4)	58.2 (± 12.3)	0.053
	Total	333	313	646	
					
**Age**	< 50	91 (52.3)	83 (47.7)	174 (100)	0.05
	50–59	108 (59.3)	74 (40.7)	182 (100)	
	60–69	80 (45.7)	95 (54.3)	175 (100)	
	≥ 70	54 (47.0)	61 (53.0)	115 (100)	
	total	333 (51.5)	313 (48.5)	646 (100)	
					
**pT**^a^	pT1	185 (56.9)	140 (43.1)	325 (100)	**0.006**
	PT2 to 4	139 (45.9)	164 (54.1)	303 (100)	
	Total	324 (51.6)	304 (48.4)	628 (100)	
					
**pN**^a^	pN0	193 (52.9)	172 (47.1)	365 (100)	0.504
	pN1	134 (50.2)	133 (49.8)	267 (100)	
	Total	327 (51.7)	305 (48.3)	632 (100)	
					
**Grading**	1 or 2	240 (55.0)	196 (45.0)	436 (100)	**0.007**
	3	90 (43.7)	116 (56.3)	206 (100)	
	Total	330 (51.4)	312 (48.6)	642 (100)	
					
**Hormone receptor Status**	Negative	54 (43.2)	71 (56.8)	125 (100)	0.177
	Positive	234 (50.0)	234 (50.0)	468 (100)	
	Total	288 (48.6)	305 (51.4)	593 (100)	

^a^According to Sobin LH, Wittekind CH: UICC: TNM Classification of Malignant Tumours. 6^th ^edition, New York: Wiley; 2002 [36]. SD, Standard deviation; n/a, not available; pT, Pathological assessment of the primary tumour; pN, Pathological assessment of the regional lymph nodes.

## Discussion

*ISG15 *is a type I interferon regulated gene that is induced as a primary response to diverse microbial and cell stress stimuli [10]. Although the biological activities of *ISG15 *have yet to be elucidated, it is thought that *ISG15 *plays an important role in host defence and stress response pathways that have antitumour functions [10]. Consistent with this view, alterations in the *ISG15 *pathway have been identified in human tumours and in tumour cell lines. Increased expression of free and conjugated *ISG15 *has been exhibited in human breast, ovarian, prostate, colorectal and melanoma tumour cell lines compared with normal cell lines [13]. In accordance with the work of Desai and colleagues [13], who analysed two cell lines and showed abundant expression of *ISG15 *in the breast cancer cell line ZR-75-1 but not in the BT474 cell line, we also found upregulation of *ISG15 *mRNA in several additional breast cancer cell lines analysed (that is, BT20, MDA-MB468, MDA-MB231, T47D and MCF7) when compared with the benign mammary cell lines HMEC and MCF12A. Interestingly, upregulation of *ISG15 *was highly abundant in the breast cancer cell line MDA-MB231, a cell line shown to be highly metastatic in nude mouse models [25]. This could implicate a possible association between *ISG15 *upregulation and enhanced proliferation or invasiveness of cancer cells.

In agreement with the findings of *ISG15 *upregulation in diverse tumour cell lines in culture, microarray analyses of human tumour biopsies have revealed enhanced expression of *ISG15 *in pancreatic adenocarcinoma [14], endometrial cancer [13] and bladder cancer [12], when compared with the respective normal tissues. Expression of *ISG15 *in human cancerous and normal breast tissue has not been previously analysed. In the current study, a systematic characterisation of *ISG15 *expression in human breast cancer cells is presented for the first time at both the mRNA and the protein level, and includes a large cohort of breast carcinoma specimens that have been analysed by correlative analysis using clinicopathological parameters and patient survival data.

In accordance with previous findings in human cancer tissue [12-14] we found upregulation of *ISG15 *at the mRNA level in breast carcinomas compared with normal breast tissue. Enhanced expression of *ISG15 *in breast cancer was further validated by cDNA dot-blot analysis. Overexpression of *ISG15 *protein in breast carcinomas could be confirmed by immunohistochemistry of a TMA. Using this technique, *ISG15 *protein expression was correlated with patient survival data. In the initial cohort of breast carcinomas (*n* = 179) we found a significant correlation between *ISG15 *expression and unfavourable prognosis (RFS; *p *= 0.012) suggesting a potential role of *ISG15 *in breast cancer development. Analysis of the second, independent breast cancer cohort (*n* = 967) confirmed this. I0n the validation TMA set, patients with *ISG15 *overexpression (IRS > 3) showed unfavourable prognosis in RFS (*p *= 0.03), EFS (*p *= 0.001) and OS analyses (*p *= 0.001). This unfavourable prognosis of patients with abundant *ISG15 *expression might be due to an insufficient response to chemotherapy in this subgroup of patients. Indeed, deregulated *ISG15 *expression has been found in response to diverse cancer chemotherapeutic agents, such as paclitaxel in the treatment of ovarian carcinomas and 5-fluorouracil in the therapy of oesophageal cancer [26,27]. Bani and colleagues [26] analysed mice with human ovarian carcinoma xenografts undergoing treatment with paclitaxel as a way of understanding the *in vivo *molecular consequences of drug treatment. By analysing expression changes by cDNA microarray they found that paclitaxel could affect the level of expression of a variety of genes in those ovarian tumours responding to treatment. So the expression of several interferon-inducible genes such as *ISG15 *was reduced in the responders. Surprisingly, in human oesophageal squamous cancer cell lines, *ISG15 *was found to be upregulated after treatment with the chemotherapeutic agent 5-fluorouracil as shown by DNA microarray analysis and reverse transcription-PCR [27]. *ISG15 *mRNA expression was also found to be upregulated after treatment of human colorectal and breast cancer cell lines with campothecin, a topoisomerase I inhibitor [28,29]. Therefore *ISG15 *may be regulated differentially in response to different chemotherapeutic regimens.

Interestingly, *ISG15 *expression was associated with ER status in the initial TMA set (*p *= 0.028; Additional file 3), but not in the validation TMA set (*p *= 0.177, Table 2), as oestrogen is known to play a modulatory role in the ISGylation pathway. Indeed the ligating (E3) enzyme, that is a part of the post-translational modification by *ISG15*, was shown to be oestrogen-responsive [30]. The so-called ISGylation is mediated by a multi-step pathway, comprised of activating (E1), conjugating (E2) and ligating (E3) enzyme components [30]. The oestrogen responsiveness of the ligating (E3) enzyme in the ISGylation pathway is of great importance because ERs play a major role in the regulation of growth, survival and differentiation of normal and malignant breast epithelial cells [31,32]. Therefore, the determination of breast tumour hormone receptor status is of major importance for therapy selection [33]. Approximately 60 to 80% of all breast cancers abundantly express ERα, but only two-thirds of those patients are responsive to endocrinal treatment (anti-oestrogen therapy). Intriguingly, a proportion of ERα-positive tumours do not respond to hormone treatment at all (*de novo *resistance) while the majority of those tumours that initially responded to anti-oestrogens eventually become resistant during treatment (acquired resistance). Most ER-resistant tumours remain ERα-positive, suggesting a continued role for ERα in breast cancer cell survival and proliferation [34,35]. The likely development of ER-resistance during breast cancer treatment with anti-oestrogens emphasises the urgent need for surrogate target molecules that may be able to bypass these resistances. Therefore, *ISG15 *might be a target molecule in the therapy of drug-resistant breast tumours.

## Conclusion

Our analyses showed *ISG15 *overexpression in human breast carcinomas relative to normal breast tissue at the RNA and protein level indicating that *ISG15 *may represent a candidate oncogene in human breast cancer. Interestingly, *ISG15 *could function as a novel breast tumour marker with prognostic or predictive significance. Recently, microarray experiments determined strong deregulation of *ISG15 *expression in response to diverse cancer chemotherapeutic agents, for example, paclitaxel in the treatment of ovarian carcinomas or 5-fluorouracil in the therapy of oesophageal cancer. These findings indicate that *ISG15 *could represent a predictive biomarker in the treatment of these cancers as well. However, the putative function of *ISG15 *in breast carcinogenesis and in chemotherapeutic response has to be further analysed in prospective studies. As a next step we will investigate whether an *ISG15*-based diagnostic assay is able to predict response to specific chemotherapy regimens in the treatment of breast cancer.

## Abbreviations

BBCC = Bavarian Breast Cancer Cases and Controls; EFS = Event-free survival; ER = Oestrogen receptor; H&E = Haematoxylin and eosin; IRS = Immunoreactivity Score; *ISG15* = Interferon-stimulated gene 15 kDa; OS = Overall survival; PCR = polymerase chain reaction; RFS = Recurrence-free survival; TMA = Tissue microarray.

## Competing interests

The authors declare that they have no competing interests.

## Authors' contributions

NB: participated in the design of the study, data analysis, data interpretation, establishment and evaluation of the immunohistochemistry and drafted the manuscript; EN: carried out the immunohistochemical studies, and critically revised the manuscript; JV: supported with expertise in molecular biology techniques and in data interpretation and critically revised the manuscript; MFP: participated in construction of the BBCC-TMA and data analysis; GK: supported in data interpretation and critically revised the manuscript; AN: participated in the evaluation and interpretation of the immunohistochemical data; AH: supported in data interpretation and critically revised the manuscript; AD: did the histological review for all tumours included in the TMA and participated in TMA construction and data analyses, and critically revised the manuscript; MWB: supported in data interpretation and critically revised the manuscript; RK: participated in design and coordination of the study, and critically revised the manuscript; PAF: participated in construction of the BBCC-TMA, study design and coordination, data analysis, data interpretation and drafting of the manuscript; ED: conceived the study, participated in the study design and coordination, molecular and data analysis, data interpretation and drafting of the manuscript.

**Figure 8:**
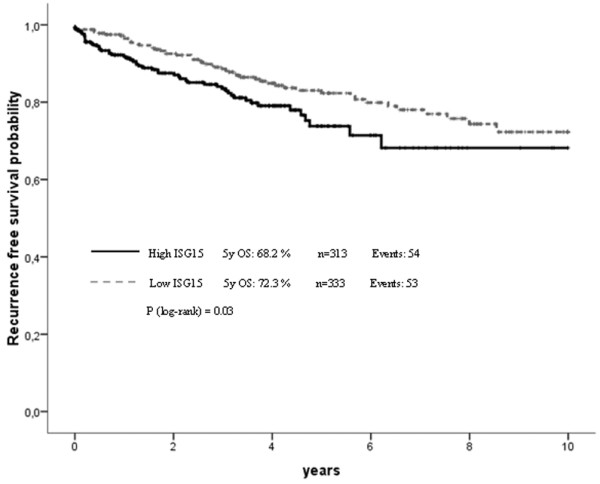
Correlation of *ISG15 *expression and patient prognosis according to univariate Kaplan-Meier analysis. Breast cancer patients overexpressing *ISG15 *have an unfavourable prognosis in recurrence-free survival analysis (*p *= 0.030).

## Supplementary Material

Additional file 1 Tif file that shows the correlation of *ISG15 *expression and patient prognosis according to univariate Kaplan-Meier analysis in the initial TMA set. Breast cancer patients expressing *ISG15 *exhibit an unfavourable prognosis in recurrence-free survival analysis (*p *= 0.012). Click here for file

Additional file 2 Tif file that shows correlation of *ISG15 *expression and patient prognosis according to univariate Kaplan-Meier analysis in the initial TMA set. Breast cancer patients expressing *ISG15 *show a trend towards unfavourable prognosis in overall survival analysis (*p *= 0.128). Click here for file

Additional file 3 Word file containing a table presenting the clinicopathological and immunohistochemical parameters in relation to *ISG15 *immunoreactivity in the initial TMA set. Click here for file

Additional file 4 Word file containing a table presenting the univariate analysis of factors regarding overall survival (OS) and recurrence-free survival (RFS) in the initial TMA set. Click here for file

## References

[B1] Loeb KR, Haas AL (1992). The interferon-inducible 15-kDa ubiquitin homolog conjugates to intracellular proteins. J Biol Chem.

[B2] Kim KI, Zhang DE (2003). *ISG15*, not just another ubiquitin-like protein. Biochem Biophys Res Commun.

[B3] Der SD, Zhou A, Williams BR, Silverman RH (1998). Identification of genes differentially regulated by interferon alpha, beta, or gamma using oligonucleotide arrays. Proc Natl Acad Sci USA.

[B4] Haas AL, Ahrens P, Bright PM, Ankel H (1987). Interferon induces a 15-kilodalton protein exhibiting marked homology to ubiquitin. J Biol Chem.

[B5] Hamerman JA, Hayashi F, Schroeder LA, Gygi SP, Haas AL, Hampson L, Coughlin P, Aebersold R, Aderem A (2002). Serpin 2a is induced in activated macrophages and conjugates to a ubiquitin homolog. J Immunol.

[B6] Malakhov MP, Kim KI, Malakhova OA, Jacobs BS, Borden EC, Zhang DE (2003). High-throughput immunoblotting. Ubiquitin-like protein *ISG15* modifies key regulators of signal transduction. J Biol Chem.

[B7] Giannakopoulos NV, Luo JK, Papov V, Zou W, Lenschow DJ, Jacobs BS, Borden EC, Li J, Virgin HW, Zhang DE (2005). Proteomic identification of proteins conjugated to *ISG15* in mouse and human cells. Biochem Biophys Res Commun.

[B8] Zhao C, Denison C, Huibregtse JM, Gygi S, Krug RM (2005). Human *ISG15* conjugation targets both IFN-induced and constitutively expressed proteins functioning in diverse cellular pathways. Proc Natl Acad Sci USA.

[B9] Liu YC, Penninger J, Karin M (2005). Immunity by ubiquitylation: a reversible process of modification. Nat Rev Immunol.

[B10] Andersen JB, Hassel BA (2006). The interferon regulated ubiquitin-like protein, *ISG15*, in tumorigenesis: friend or foe?. Cytokine Growth Factor Rev.

[B11] Polyak K, Xia Y, Zweier JL, Kinzler KW, Vogelstein B (1997). A model for p53-induced apoptosis. Nature.

[B12] Andersen JB, Aaboe M, Borden EC, Goloubeva OG, Hassel BA, Orntoft TF (2006). Stage-associated overexpression of the ubiquitin-like protein, *ISG15*, in bladder cancer. Br J Cancer.

[B13] Desai SD, Haas AL, Wood LM, Tsai YC, Pestka S, Rubin EH, Saleem A, Nur-E-Kamal A, Liu LF (2006). Elevated expression of *ISG15 *in tumor cells interferes with the ubiquitin/26S proteasome pathway. Cancer Res.

[B14] Iacobuzio-Donahue CA, Maitra A, Olsen M, Lowe AW, van Heek NT, Rosty C, Walter K, Sato N, Parker A, Ashfaq R, Jaffee E, Ryu B, Jones J, Eshleman JR, Yeo CJ, Cameron JL, Kern SE, Hruban RH, Brown PO, Goggins M (2003). Exploration of global gene expression patterns in pancreatic adenocarcinoma using cDNA microarrays. Am J Pathol.

[B15] Pitha-Rowe IF, Pitha PM (2007). Viral defense, carcinogenesis and *ISG15*: novel roles for an old ISG. Cytokine Growth Factor Rev.

[B16] Jurica MS, Moore MJ (2003). Pre-mRNA splicing: awash in a sea of proteins. Mol Cell.

[B17] Dahl E, Kristiansen G, Gottlob K, Klaman I, Ebner E, Hinzmann B, Hermann K, Pilarsky C, Durst M, Klinkhammer-Schalke M, Blaszyk H, Knuechel R, Hartmann A, Rosenthal A, Wild PJ (2006). Molecular profiling of laser-microdissected matched tumor and normal breast tissue identifies karyopherin alpha2 as a potential novel prognostic marker in breast cancer. Clin Cancer Res.

[B18] Elston CW, Ellis IO (1991). Pathological prognostic factors in breast cancer. The value of histological grade in breast cancer: experience from a large study with long-term follow-up. Histopathology.

[B19] Fasching PA, Loehberg CR, Strissel PL, Lux MP, Bani MR, Schrauder M, Geiler S, Ringleff K, Oeser S, Weihbrecht S, Schulz-Wendtland R, Hartmann A, Beckmann MW, Strick R (2007). Single nucleotide polymorphisms of the aromatase gene *(CYP19A1)*, HER2/neu status, and prognosis in breast cancer patients. Breast Cancer Res Treat.

[B20] Schrauder M, Frank S, Strissel PL, Lux MP, Bani MR, Rauh C, Sieber CC, Heusinger K, Hartmann A, Schulz-Wendtland R, Strick R, Beckmann MW, Fasching PA (2008). Single nucleotide polymorphism D1853N of the ATM gene may alter the risk for breast cancer. J Cancer Res Clin Oncol.

[B21] Zafrakas M, Chorovicer M, Klaman I, Kristiansen G, Wild PJ, Heindrichs U, Knüchel R, Dahl E (2006). Systematic characterisation of GABRP expression in sporadic breast cancer and normal breast tissue. Int J Cancer.

[B22] Veeck J, Niederacher D, An H, Klopocki E, Wiesmann F, Betz B, Galm O, Camara O, Durst M, Kristiansen G, Huszka C, Knuchel R, Dahl E (2006). Aberrant methylation of the Wnt antagonist SFRP1 in breast cancer is associated with unfavourable prognosis. Oncogene.

[B23] Clontech/Information on the Cancer Profiling Array. http://www.clontech.com/images/pt/PT3140-1.pdf.

[B24] Remmele W, Stegner HE (1987). Recommendation for uniform definition of an immunoreactive score (IRS) for immunohistochemical estrogen receptor detection (ER-ICA) in breast cancer tissue. Pathologe.

[B25] Gallagher SM, Castorino JJ, Wang D, Philp NJ (2007). Monocarboxylate transporter 4 regulates maturation and trafficking of CD147 to the plasma membrane in the metastatic breast cancer cell line MDA-MB-231. Cancer Res.

[B26] Bani MR, Nicoletti MI, Alkharouf NW, Ghilardi C, Petersen D, Erba E, Sausville EA, Liu ET, Giavazzi R (2004). Gene expression correlating with response to paclitaxel in ovarian carcinoma xenografts. Mol Cancer Ther.

[B27] Matsumura Y, Yashiro M, Ohira M, Tabuchi H, Hirakawa K (2005). 5-Fluorouracil up-regulates interferon pathway gene expression in esophageal cancer cells. Anticancer Res.

[B28] Liu M, Hummer BT, Li X, Hassel BA (2004). Camptothecin induces the ubiquitin-like protein, *ISG15*, and enhances *ISG15* conjugation in response to interferon. J Interferon Cytokine Res.

[B29] Desai SD, Mao Y, Sun M, Li TK, Wu J, Liu LF (2000). Ubiquitin, SUMO-1, and UCRP in camptothecin sensitivity and resistance. Ann N Y Acad Sci.

[B30] Horie-Inoue K, Inoue S (2006). Epigenetic and proteolytic inactivation of 14-3-3sigma in breast and prostate cancers. Semin Cancer Biol.

[B31] Khan SA, Rogers MA, Khurana KK, Meguid MM, Numann PJ (1998). Estrogen receptor expression in benign breast epithelium and breast cancer risk. J Natl Cancer Inst.

[B32] Ricketts D, Turnbull L, Ryall G, Bakhshi R, Rawson NS, Gazet JC, Nolan C, Coombes RC (1991). Estrogen and progesterone receptors in the normal female breast. Cancer Res.

[B33] Moy B, Goss PE (2006). Estrogen receptor pathway: resistance to endocrine therapy and new therapeutic approaches. Clin Cancer Res.

[B34] Coombes RC, Powles TJ, Berger U, Wilson P, McClelland RA, Gazet JC, Trott PA, Ford HT (1987). Prediction of endocrine response in breast cancer by immunocytochemical detection of oestrogen receptor in fine-needle aspirates. Lancet.

[B35] Taylor RE, Powles TJ, Humphreys J, Bettelheim R, Dowsett M, Casey AJ, Neville AM, Coombes RC (1982). Effects of endocrine therapy on steroid-receptor content of breast cancer. Br J Cancer.

[B36] Sobin LH, Wittekind CH (2002). UICC: TNM Classification of Malignant Tumours.

