# Supplementary figures and images for: The ubiquitin-like molecule interferon-stimulated gene 15 (ISG15) is a potential prognostic marker in human breast cancer
Source: Breast Cancer Res. 2008 Jul 15;10(4):R58. doi: 10.1186/bcr2117 (PMC2575531; doi:10.1186/bcr2117)

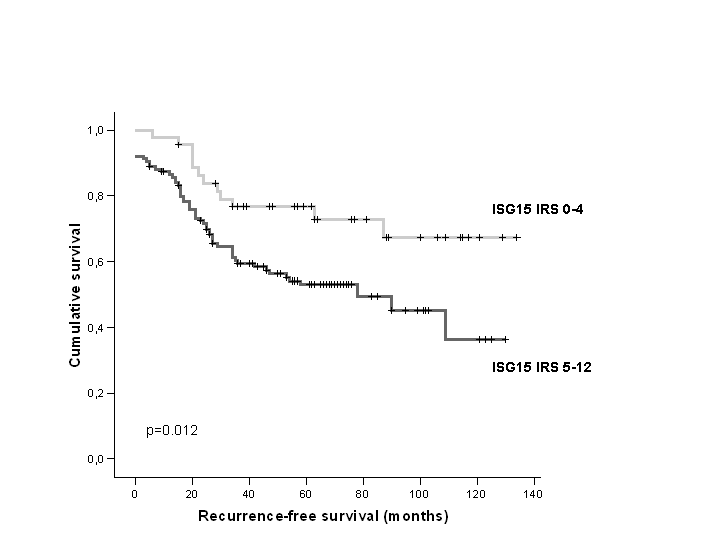

Supplement: Additional file 1 — Tif file that shows the correlation of ISG15 expression and patient prognosis according to univariate Kaplan-Meier analysis in the initial TMA set. Breast cancer patients expressing ISG15 exhibit an unfavourable prognosis in recurrence-free survival analysis (p = 0.012). [file bcr2117-S1.tiff]

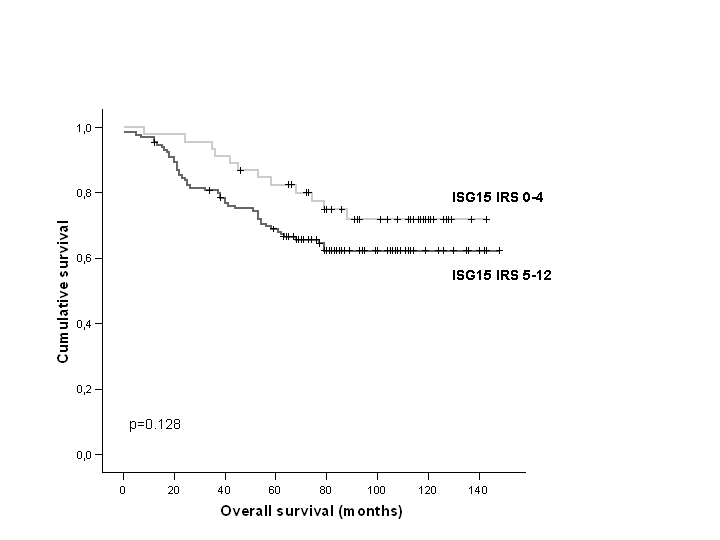

Supplement: Additional file 2 — Tif file that shows correlation of ISG15 expression and patient prognosis according to univariate Kaplan-Meier analysis in the initial TMA set. Breast cancer patients expressing ISG15 show a trend towards unfavourable prognosis in overall survival analysis (p = 0.128). [file bcr2117-S2.tiff]
