# Supplementary material for: The ubiquitin-like molecule interferon-stimulated gene 15 (ISG15) is a potential prognostic marker in human breast cancer
Source: Breast Cancer Res. 2008 Jul 15;10(4):R58. doi: 10.1186/bcr2117 (PMC2575531; doi:10.1186/bcr2117)
Supplement: Additional file 3 — Word file containing a table presenting the clinicopathological and immunohistochemical parameters in relation to ISG15 immunoreactivity in the initial TMA set. [file bcr2117-S3.doc]

| **Table 1.** Clinicopathological and immunohistochemical parameters | | | | | |
| --- | --- | --- | --- | --- | --- |
| in relation to ISG15 immunoreactivity | | | | | |
|  |  |  |  |  |  |
| **Variable** | **Categorisation** | **ISG15 immunoreactivity** | | | |
|  |  | **n analysable** | **negative**^b^ | **positive**^b^ | **p**^c^ |
|  |  |  |  |  |  |
| ***Clinicopathological data:*** | |  |  |  |  |
| Tumour stage^a^ | |  |  |  |  |
|  | pT1 | 48 | 17 | 31 | 0.213 |
|  | pT2 | 88 | 22 | 66 |  |
|  | pT3 | 11 | 3 | 8 |  |
|  | pT4 | 29 | 4 | 25 |  |
| Lymph node status^a^ | |  |  |  |  |
|  | pN0 | 73 | 23 | 50 | 0.159 |
|  | pN1-3 | 98 | 23 | 75 |  |
| Histologic grade | |  |  |  |  |
|  | G1 | 17 | 6 | 11 | 0.160 |
|  | G2 | 80 | 25 | 55 |  |
|  | G3 | 79 | 15 | 64 |  |
| Multifocality | |  |  |  |  |
|  | unifocal tumour | 150 | 40 | 110 | 0.628 |
|  | multifocal tumour | 27 | 6 | 21 |  |
| Histologic type | |  |  |  |  |
|  | Ductal | 141 | 34 | 107 | 0.484 |
|  | Lobular | 14 | 5 | 9 |  |
|  | Other | 18 | 6 | 12 |  |
|  |  |  |  |  |  |
| ***Immunohistochemistry (IHC):*** | |  |  |  |  |
| Oestrogen receptor status | |  |  |  |  |
|  | Negative | 52 | 19 | 33 | **0.028** |
|  | Positive | 94 | 18 | 76 |  |
| Progesterone receptor status | |  |  |  |  |
|  | Negative | 108 | 25 | 83 | 0.418 |
|  | Positive | 46 | 14 | 32 |  |
| HER2 status | |  |  |  |  |
|  | weak (0-2+) | 129 | 36 | 93 | 0.347 |
|  | strong (3+) | 28 | 5 | 23 |  |
|  |  |  |  |  |  |
| ^a^According to UICC: TNM Classification of Malignant Tumours. 6^th^ edn (2002) Sobin  LH, Wittekind CH (eds) Wiley: New York [36] | | | | | |
| ^b^ISG15 immunoreactivity: negative=IRS 0-4, positive=IRS 5-12 | | | | | |
| ^c^Fisher's exact test (two-sided), bold face representing significant data (*P* < 0.05) | | | | | |
