# Supplementary material for: The ubiquitin-like molecule interferon-stimulated gene 15 (ISG15) is a potential prognostic marker in human breast cancer
Source: Breast Cancer Res. 2008 Jul 15;10(4):R58. doi: 10.1186/bcr2117 (PMC2575531; doi:10.1186/bcr2117)
Supplement: Additional file 4 — Word file containing a table presenting the univariate analysis of factors regarding overall survival (OS) and recurrence-free survival (RFS) in the initial TMA set. [file bcr2117-S4.doc]

| **Table 2.** Univariate analysis of factors regarding | | | | | | | | | | |
| --- | --- | --- | --- | --- | --- | --- | --- | --- | --- | --- |
| overall survival (OS) and recurrence-free survival (RFS) | | | | | | | | | | |
|  |  |  | | |  | | |  | |  |
| **Variable** | **Categorisation** | **Tumour-related death (OS)** | | | | **Tumour recurrence (RFS)** | | | | |
|  |  | **n** | **events** | **p**^c^ | | **n** | **events** | | **p**^c^ | |
|  |  |  |  |  | |  |  | |  | |
| ***Clinicopathological data:*** | |  |  |  | |  |  | |  | |
| Tumour stage^a^ | |  |  |  | |  |  | |  | |
|  | pT1 | 48 | 7 | **<0.0001** | | 47 | 7 | | **<0.0001** | |
|  | pT2 | 88 | 31 |  |  | 86 | 40 | |  |  |
|  | pT3 | 11 | 4 |  |  | 10 | 4 | |  |  |
|  | pT4 | 29 | 18 |  |  | 26 | 19 | |  |  |
| Lymph node status^a^ | |  |  |  | |  |  | |  | |
|  | pN0 | 73 | 11 | **<0.0001** | | 72 | 13 | | **<0.0001** | |
|  | pN1-3 | 98 | 45 |  |  | 94 | 53 | |  |  |
| Histologic grade | |  |  |  | |  |  | |  | |
|  | G1 | 17 | 3 | **0.001** | | 17 | 3 | | **<0.0001** | |
|  | G2 | 80 | 20 |  |  | 75 | 25 | |  |  |
|  | G3 | 79 | 37 |  |  | 77 | 41 | |  |  |
| Multifocality | |  |  |  | |  |  | |  | |
|  | unifocal tumour | 150 | 50 | 0.630 | | 145 | 60 | | 0.964 | |
|  | multifocal tumour | 27 | 10 |  |  | 25 | 10 | |  |  |
| Histologic type | |  |  |  | |  |  | |  | |
|  | Ductal | 141 | 46 | 0.525 | | 138 | 58 | | 0.635 | |
|  | Lobular | 14 | 7 |  |  | 12 | 5 | |  |  |
|  | Other | 18 | 6 |  |  | 16 | 5 | |  |  |
|  |  |  |  |  | |  |  | |  | |
| ***Immunohistochemistry (IHC):*** | |  |  |  | |  |  | |  | |
| Oestrogen receptor status | |  |  |  | |  |  | |  | |
|  | Negative | 52 | 20 | 0.201 | | 52 | 24 | | 0.262 | |
|  | Positive | 94 | 28 |  |  | 90 | 32 | |  |  |
| Progesterone receptor status | |  |  |  | |  |  | |  | |
|  | Negative | 108 | 45 | **0.009** | | 102 | 48 | | **0.013** | |
|  | Positive | 46 | 9 |  |  | 46 | 11 | |  |  |
| HER2 Status | |  |  |  | |  |  | |  | |
|  | weak (0-2+) | 129 | 37 | **0.004** | | 122 | 46 | | 0.058 | |
|  | strong (3+) | 28 | 15 |  |  | 28 | 15 | |  |  |
| ISG15^b^ |  |  |  |  | |  |  | |  | |
|  | Negative (IRS 0-4) | 46 | 12 | 0.128 | | 44 | 12 | | **0.012** | |
|  | Positive (IRS 5-12) | 131 | 48 |  |  | 126 | 58 | |  |  |
|  |  |  |  |  | |  |  | |  | |
| ^a^According to UICC: TNM Classification of Malignant Tumours. 6^th^ edn (2002) Sobin LH, Wittekind CH (eds) Wiley: New York [36] | | | | | | | | | | |
| ^b^ISG15 immunoreactivity: negative=IRS 0-4, positive=IRS 5-12 | | | | | | | | | | |
| ^c^Log-rank test (two-sided), bold face representing significant data (*P* < 0.05) | | | | | | | | | | |
